# GSTM1 and GSTT1 polymorphisms are associated with increased bladder cancer risk: Evidence from updated meta-analysis

**DOI:** 10.18632/oncotarget.13702

**Published:** 2016-11-29

**Authors:** Cui Yu, Chen Hequn, Liu Longfei, Wang Long, Chen Zhi, Zeng Feng, Chen Jinbo, Li Chao, Zu Xiongbing

**Affiliations:** ^1^ Department of Urology, Xiangya Hospital, Central South University, Changsha, Hunan 410000, China

**Keywords:** GSTM1, GSTT1, polymorphism, bladder cancer

## Abstract

**Background:**

Previous studies have indicated association between *GSTM1* and *GSTT1* gene polymorphisms and bladder cancer susceptibility, but the results have been inconclusive. Here, we performed a meta-analysis to investigate the association between *GSTM1*/*GSTT1* deletion polymorphisms and bladder cancer susceptibility.

**Methods:**

We searched for all studies investigating the association between GSTM1 or GSTT1 polymorphism and bladder cancer susceptibility in Pubmed, Web of Knowledge, and the Cochrane Central Search Library. A systematic review and meta-analysis were performed. Subgroup analyses were performed on different ethnicity, population-based and smoking status.

**Results:**

Our search identified 63 studies. *GSTM1* null, *GSTT1* null and *GSTM1*/*GSTT1* double-null genotypes were associated with increased risk of bladder cancer (OR: 1.36 95% CI: 1.25-1.47, P<0.01; OR: 1.13 95% CI: 1.02-1.25, P<0.01; OR: 1.84 95% CI: 1.50-2.26, P<0.01). Subgroup analyses indicated that the *GSTM1*-null genotype was associated with increased risk of bladder cancer in Caucasians and Asians, while the *GSTT1*-null genotype was associated with increased risk of bladder cancer in Caucasians. The *GSTM1*/*GSTT1* double-null genotype was associated with increased risk of bladder cancer in Caucasians, Asians, and Africans. Stratified analyses of population-based associations indicated increased bladder cancer risk associated with *GSTM1*-null and *GSTM1*/*GSTT1* double-null genotypes in hospital-based and population-based studies. *GSTM1* deletion was associated with increased bladder cancer risk in both smokers and nonsmokers. Non-smokers with the *GSTM1*/*GSTT1* double-null genotype had an increased bladder cancer risk.

**Conclusion:**

This meta-analysis demonstrates that the *GSTM1*-null, *GSTT1*-null, and *GSTM1*/*GSTT1* double-null genotypes are associated with increased bladder cancer risk.

## INTRODUCTION

Bladder cancer is the ninth most common malignancy worldwide, and the fourth most common malignancy in the United States [1, 2]. 70% of bladder cancers are nonmuscle invasive, whereas the remaining 30% are muscle invasive bladder cancers [3]. Life style and occupational exposure are the main etiological factors in bladder cancer [4]. However, only a small percentage of people develop bladder cancer after exposure to these environmental factors, indicating that genetic susceptibility plays an important role in bladder cancer development.

Glutathione S-transferases (GSTs) are members of a multigene family of phase II enzymes, which are involved in the detoxification of various carcinogens, and have been recognized as an important factor in bladder cancer development [5]. *GSTM1* and *GSTT1* genes are members of the GST family. *GSTM1* and *GSTT1* homozygous deletions are associated with reduced detoxification function, increased susceptibility to cytogenetic damage, and increased risk of cancer [6–8]. Previous meta-analyses have indicated an association of *GSTM1* and *GSTT1* deletion polymorphisms with increased bladder cancer risk [9–11]; however, the results have been inconsistent. Some overlapping studies were not excluded and several published studies were missing in their analysis [12, 13]. Here, we performed an updated meta-analysis to investigate the association between *GSTM1/GSTT1* deletion polymorphisms and bladder cancer susceptibility.

## RESULTS

**Figure 1:**
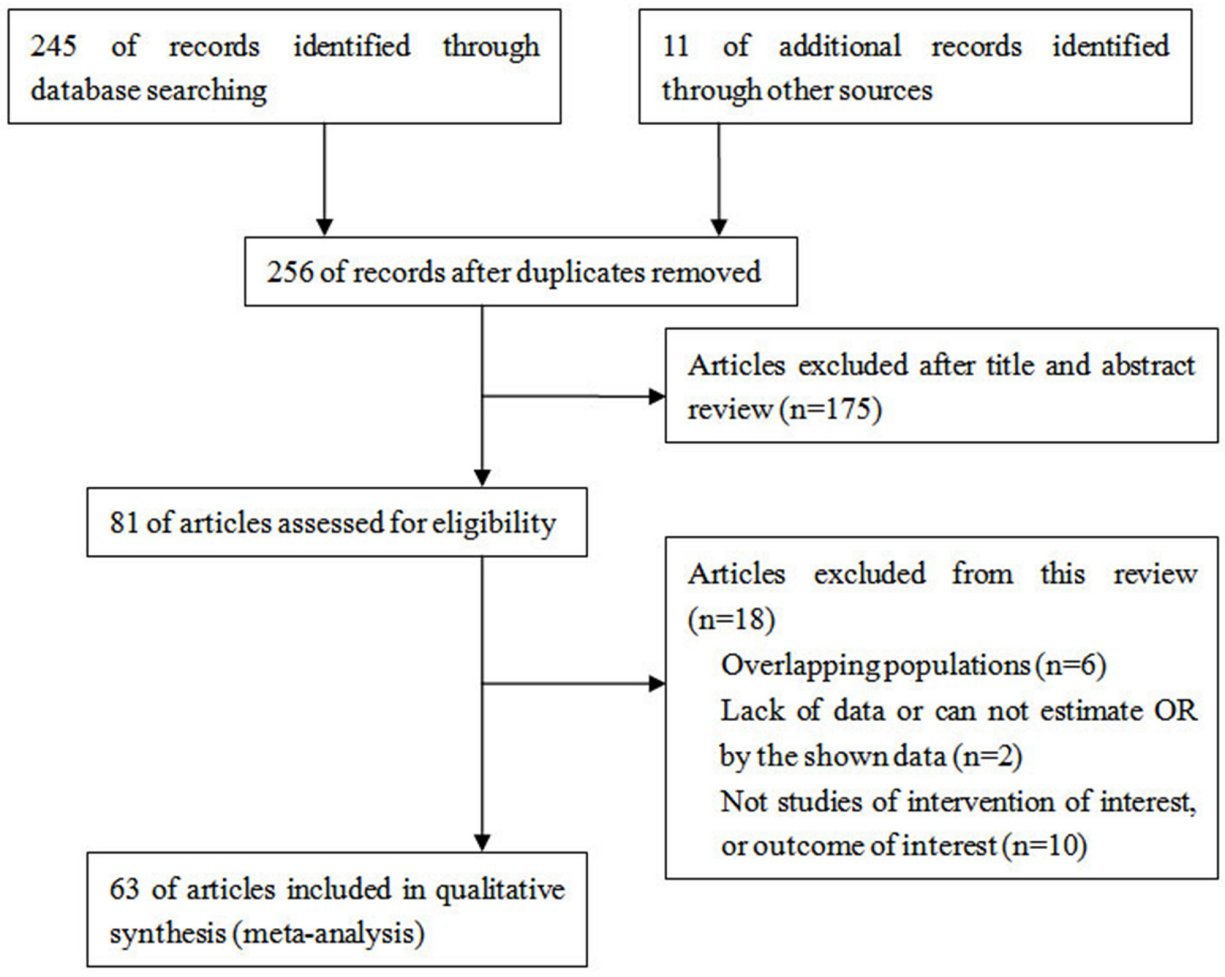
Flow chart of article selection

256 studies were identified from the database or manual search. According to the selection criteria, 193 studies were excluded, resulting in 63 studies for analysis [5, 12, 14–75]. (Figure 1) From these studies, data were available from 46 studies on GSTM1 null genotype (12751 cases and 15519 controls), 54 studies on GSTT1 null genotype (11817 cases and 14805 controls) and 11 studies on GSTM1/GSTT1 double-null genotype (1485 cases and 2230 controls). The essential information of the included studies is listed in Table 1.

**Table 1:** Characteristics of patients in the studies included

Study	Year	Ethnicity	Source of controls	Study quality	GSMT1 null		GSTT1 null		GSTM1/ GSTT1 null	
					case^a^	contro^a^	case^a^	contro^a^	case^a^	contro^a^
Ceylan	2015	Caucasian	HB	9	22/43	31/39	19/46	9/61	8/57	8/62
Matic	2014	Caucasian	HB	8	80/63	56/58	36/101	32/82		
Reszka	2014	Caucasian	PB	9	149/95	165/200	30/212	77/288		
Berber	2013	Caucasian	PB	8	54/60	51/63	31/83	16/98	11/103	7/107
Kang	2013	Asian	PB	8	65/45	103/117	64/46	128/92		
Henríquez-Hernández	2012	Caucasian	HB	8	23/67	17/64	60/30	40/41	17/73	8/73
Schwender	2012	Caucasian	HB	9	909/663	863/876				
Lesseur	2012	Caucasian	HB	7	378/275	508/420	106/556	143/780		
Zhang	2012	Caucasian	HB	9	381/329	402/380				
Ovsiannikov	2012	Caucasian	HB	6	102/94	122/113	33/163	47/188		
Marenne	2012	Caucasian	HB	7	488/285	402/357				
Öztürk	2011	Caucasian	PB	9	98/78	51/46				
Rouissi	2011	African	HB	8	63/62	56/69	30/95	38/87		
Salinas-Sánchez	2011	Caucasian	HB	8			42/148	25/138		
Moore	2011	Caucasian	PB	9			210/794	237/942		
Goerlitz	2011	Caucasian	PB	7			147/470	156/464		
Cantor	2010	Caucasian	HB	6			136/542	160/550		
Lin	2009	Caucasian	PB	8	312/292	286/324				
Altayli	2009	Caucasian	HB	7	58/77	65/63	31/104	9/119		
Grando	2009	Multiracial	PB	8	40/60	33/67				
Song	2009	Asian	HB	9	131/77	108/104	110/98	105/107	77/131	50/162
Zupa	2009	Caucasian	PB	7	13/10	68/53				
Covolo	2008	Caucasian	HB	8	17/9	14/16	42/155	33/178		
Golka	2008	Caucasian	HB	7	184/109	88/88				
Shao	2008	Asian	HB	8	85/117	81/191				
Cengiz	2007	Caucasian	HB	9	18/33	11/42	18/99	11/42		
Murta-Nascimento	2007	Caucasian	HB	8	428/251	367/368				
Zhao	2007	Caucasian	HB	9	324/298	317/316	103/520	519/115		
Kellen	2007	Caucasian	PB	7			30/164	61/319		
McGrath	2006	Multiracial	HB	8	109/161	483/439	35/156	148/776	18/173	78/844
Kogevinas	2006	Caucasian	HB	7			24/75	17/74		
García-Closas	2005	Caucasian	HB	7	716/422	571/561	230/916	248/889		
Karagas	2005	Multiracial	PB	9	163/115	211/140	53/301	83/458		
Kellen	2005	Caucasian	PB	7	312/267	597/466				
Kim	2005	Asian	HB	8	92/61	73/80	71/82	89/64		
Sobti	2005	Asian	PB	7	37/63	24/52	30/70	11/65		
Srivastava	2005	Asian	PB	9	43/63	140/230				
Broberg	2005	Caucasian	PB	8			7/54	22/132		
Saad	2005	Caucasian	PB	9			26/46	14/67		
Hung	2004	Caucasian	HB	8	132/69	112/102	43/158	33/181	28/173	19/195
Moore	2004	Multiracial	PB	7	54/52	49/60	17/89	12/97	9/97	6/103
Srivastava	2004	Asian	HB	7	42/64	54/128	28/78	29/153	16/90	9/173
Sanyal	2004	Caucasian	PB	7			66/204	12/110		
Chen	2004	Asian	PB	8			32/30	51/30		
Jeong	2003	Asian	HB	6	75/51	99/105	68/58	113/91		
Gago-Dominguez	2003	Multiracial	PB	7			50/146	34/142		
Giannakopoulos	2002	Caucasian	HB	9	56/33	56/91	5/84	16/131		
Lee	2002	Asian	HB	8	149/83	86/79	135/97	85/80	83/149	37/128
Ma	2002	Asian	PB	9	180/137	99/83	29/32	88/94		
Kim	2002	Asian	PB	8			91/125	228/221		
Aktas	2001	Caucasian	HB	6	56/47	70/132				
Törüner	2001	Caucasian	PB	7	75/46	55/66	24/97	21/100		
Kim	2000	Asian	HB	7	78/34	123/97	47/65	101/119		
Schnakenberg	2000	Caucasian	PB	8	93/64	129/94	28/129	48/175		
Steinhoff	2000	Caucasian	HB	7	80/55	57/70	20/115	17/110	12/123	4/123
Peluso	2000	Caucasian	HB	7			14/108	6/48		
Salagovic	1999	Caucasian	PB	9	40/36	123/125	21/55	42/206		
Lee	1999	Asian	HB	6			93/65	66/65		
Abdel-Rahman	1998	African	PB	7	26/11	15/19	17/20	5/29	14/23	3/31
Katoh	1998	Asian	PB	7			46/66	53/59		
Salagovic	1998	Caucasian	PB	8			20/47	42/206		
Brockmoller	1996	Caucasian	HB	6	218/156	202/171				
Kempkes	1996	Caucasian	PB	7			20/93	31/139		

a Null/present.

Abbreviations: HB, hospital-based controls; PB, population-based controls.

### GSTM1

**Figure 2:**
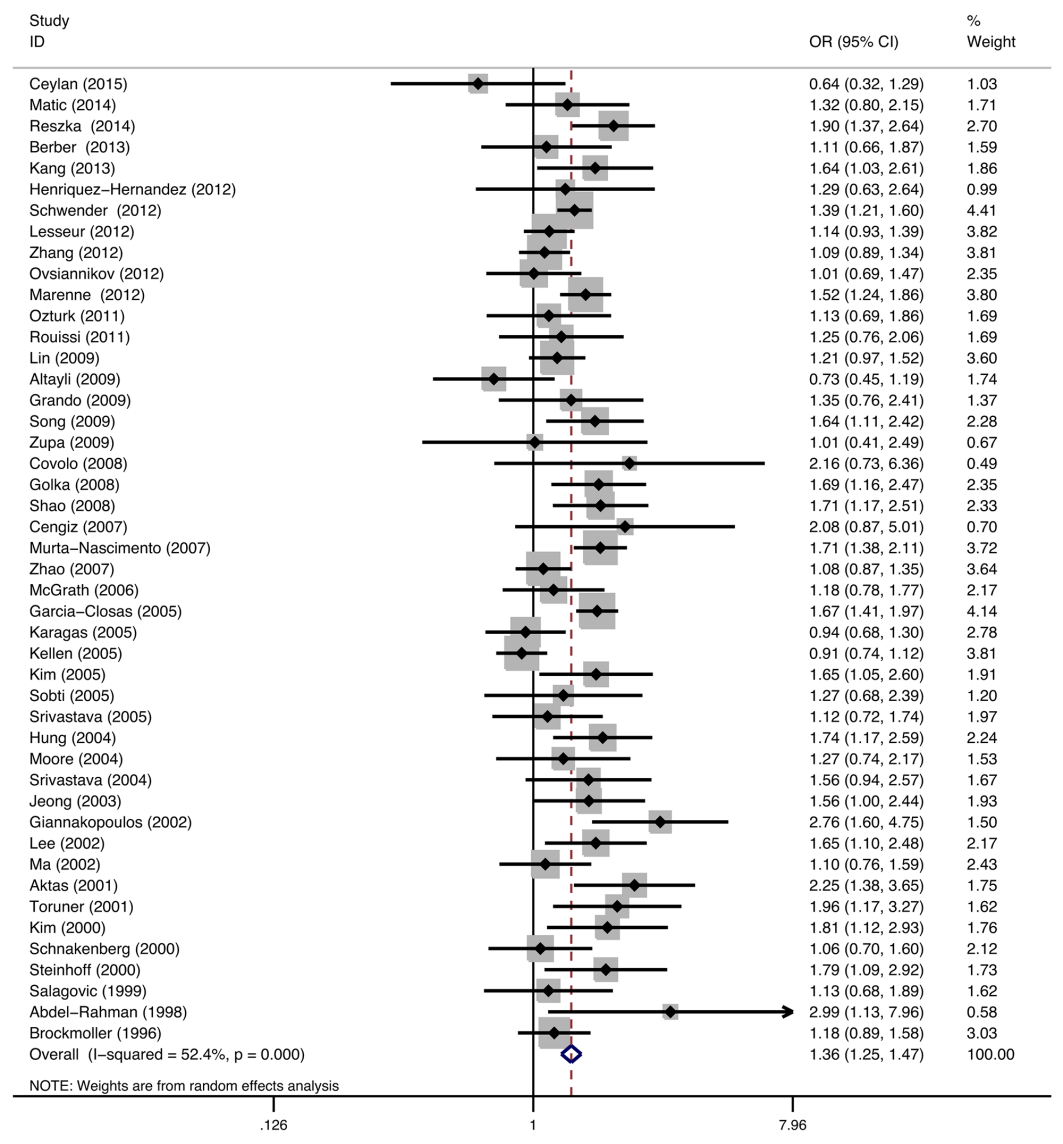
Meta-analysis of GSTM1 gene polymorphism and bladder cancer risk

46 studies described the relationship between GSTM1 polymorphism and bladder cancer susceptibility, involving 28270 individuals. Statistical heterogeneity between trials was observed in the analysis (I^2^=52.4%, P<0.01); thus, a random-effects model was used. The pooled meta-analysis showed that the GSTM1 null genotype was associated with increased risk of bladder cancer. The pooled summary of the OR was 1.36 (95% CI: 1.25-1.47, P<0.01) (Figure 2). Subgroup analyses were performed on the different ethnicity, population-based and smoking (Table 2). The GSTM1 null genotype was associated with the elevated risk of bladder cancer in Caucasians (OR=1.34, 95%CI=1.21-1.48) and Asians (OR=1.50, 95%CI=1.31-1.71). Stratified analyses of population-based association showed a significant association of elevated bladder cancer risk with GSTM1 deletion in hospital-based (HB) studies (OR=1. 42, 95%CI=1.30-1.56) and population-based (PB) studies (OR=1.22, 95%CI=1.07-1.40). The GSTM1 null genotype was also associated with elevated risk of bladder cancer stratified by smoking status (OR 1.37, 95%CI: 1.19-1.59 for smokers and OR 1.26, 95%CI: 1.08-1.48 for non-smokers, respectively).

**Table 2:** Subgroup analysis of GSTM1/GSTT1 polymorphism and bladder cancer risk

Subgroup analysis	Number	GSTM1			Number	GSTT1			Number	GSTM1/GSTT1		
Ethnicity		Null percentage (%)^#^	OR(95%CI)	Heterogeneity		Null percentage (%)^#^	OR(95%CI)	Heterogeneity		Null percentage (%)^#^	OR(95%CI)	Heterogeneity
Caucasian	29	57/50	1.34(1.21-1.48) P<0.01	p<0.01;I^2^=63.9%	38	22/19	1.23(1.08-1.40) P<0.01	P<0.01; I^2^=61.0%	5	13/7	1.77(1.20-2.60) P<0.01	p=0. 76; I^2^=0%
Asians	11	55/43	1.50(1.31-1.71) P<0.01	p=0.77; I^2^=0%	10	19/52	0.88(0.75-1.04) P=0.14	p=0.25; I^2^=21.1%	3	32/17	2.05(1.53-2.74) P<0.01	p=0. 46; I^2^=0%
Africans	2	55/45	1.74(0.76-3.99) p=0.19	p=0.12; I^2^=59%	2	30/36	0.79(0.52-1.22) P=0.29	p=0.61; I^2^=0%	1	38/9	6.29(1.62-24.47) P<0.01	-
multiracial	4	43/30	0.10(0.89-1.36) p=0.38	p=0.62; I^2^=0%	4	22/17	0.13(0.90-1.42) P=0.09	p=0.18; I^2^=36.3%	2	9/8	1.21(0.75-1.95) P=0.43	p=0. 57; I^2^=0%
HB	29	57/46	1.42(1.30-1.56) P<0.01	p<0.01; I^2^=52%	25	26/26	1.11(0.97-1.27) P=0.14	P<0.01; I^2^=48.8%	8	21/11	1.73(1.45-2.23) P<0.01	p=0. 42; I^2^=0.9%
PB	17	54/48	1.22(1.07-1.40) P<0.01	p=0.05; I^2^=39.3%	29	24/22	1.15(1.00-1.33) P=0.06	P<0.01; I^2^=62.2%	3	13/6	2.28(1.22-4.25) P=0.01	p=0. 22; I^2^=33.7%
Smoker	17	59/51	1.37(1.19-1.59) P<0.01	p=0.04; I^2^=42.5%	16	25/23	1.05(0.93-1.19) P=0.40	p=0.17; I^2^=42.5%	1	38/20	1.18 (0.54–2.59) P=0.68	-
Non-smoker	17	54/49	1.26(1.08-1.48) P<0.01	p=0.65; I^2^=0%	16	24/23	1.07(0.88-1.29) P=0.49	p=0.33; I^2^=10.4%	1	35/26	2.66 (1.22–5.81)P=0.01	-

# null genotype percentage of bladder cancer patients/ null genotype percentage of control individuals.

### GSTT1

**Figure 3:**
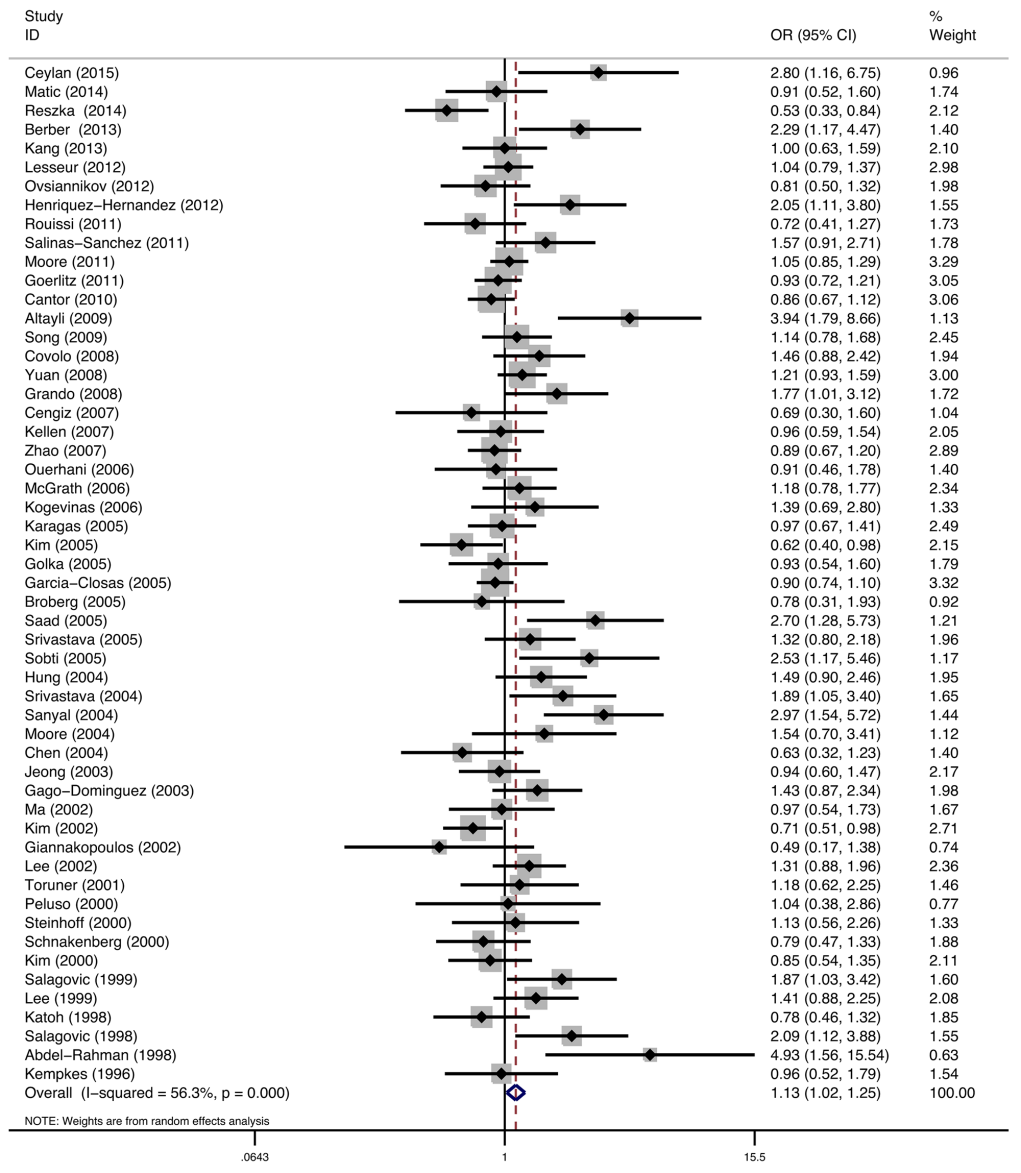
Meta-analysis of GSTT1 gene polymorphism and bladder cancer risk

54 studies described the relationship between GSTT1 polymorphism and bladder cancer susceptibility, involving 26622 individuals. Statistical heterogeneity between trials was observed in the analysis (I^2^=56.3%, P<0.01); thus, a random-effects model was used. The pooled meta-analysis showed that the GSTT1 null genotype was associated with elevated risk of bladder cancer. The pooled summary of the OR was 1.13 (95% CI: 1.02-1.25, P<0.01) (Figure 3). Subgroup analyses were performed on the different ethnicity, population-based and smoking (Table 2). The results suggested that the GSTT1 null genotype was associated with the elevated risk of bladder cancer in Caucasians (OR=1.23, 95%CI=1.08-1.40). However, no significant association was found in Asians, Africans, and multiracial subjects. Stratified analyses of population-based association showed a weak association of elevated bladder cancer risk with GSTT1 deletion in HB studies (OR=1.11, 95%CI=0.98-1.27) and PB studies (OR=1.15, 95%CI=1.00-1.33), but without statistical significance. There was no significant association between GSTM1 null genotype and bladder cancer risk stratified by smoking status (OR 1.05, 95%CI: 0.93-1.19 for smokers and OR 1.07, 95%CI: 0.88-1.29 for nonsmokers).

### GSTM1/ GSTT1

**Figure 4:**
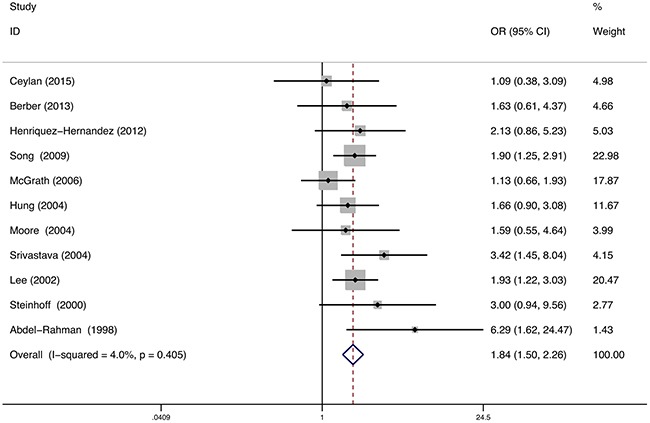
Meta-analysis of GSTM1/GSTT1 double-null gene polymorphism and bladder cancer risk

11 studies reported the relationship between GSTM1/ GSTT1 double-null and bladder cancer susceptibility, involving 3715 individuals. Statistical heterogeneity between trials was not observed in the analysis (I^2^=4%, P=0.41), so a fixed-effects model was used. The pooled meta-analysis showed that individuals with GSTM1/ GSTT1 double-null genotype were at a higher risk to develop bladder cancer than individuals with GSTM1 or GSTT1 present. The pooled summary of the OR was 1.84 (95% CI: 1.50-2.26, P<0.01) (Figure 4). Subgroup analyses were performed on the different ethnicity, population-based and smoking (Table 2). The GSTM1/ GSTT1 double-null genotype was associated with the elevated risk of bladder cancer in Caucasians (OR=1.23, 95%CI=1.08-1.40), Asians (OR=2.05, 95%CI=1.53-2.74) and Africans (OR=6.29, 95%CI=1.62-24.47). Stratified analyses of population-based association showed association of elevated bladder cancer risk with GSTM1/ GSTT1 double-null in HB studies (OR=1.73, 95%CI=1.45-2.23) and PB studies (OR=2.28, 95%CI=1.22-4.25). Only one study reported the relationship between GSTM1/ GSTT1 double-null genotype and the risk of bladder cancer stratified by smoking status; this study showed that non-smokers with GSTM1/ GSTT1 double-null genotype had an elevated bladder cancer risk (OR=2.66, 95%CI: 1.22–5.81).

### Sensitivity analysis

Sensitivity analysis was performed to assess the stability of this meta-analysis result. The influence of the individual datasets on the summary ORs was examined by repeating the meta-analysis after sequentially omitting each study. For GSTM1 null, GSTT1 null, and GSTM1/GSTT1 double-null genotypes, the ORs were not significantly affected by omitting any individual study.

### Publication bias

**Figure 5:**
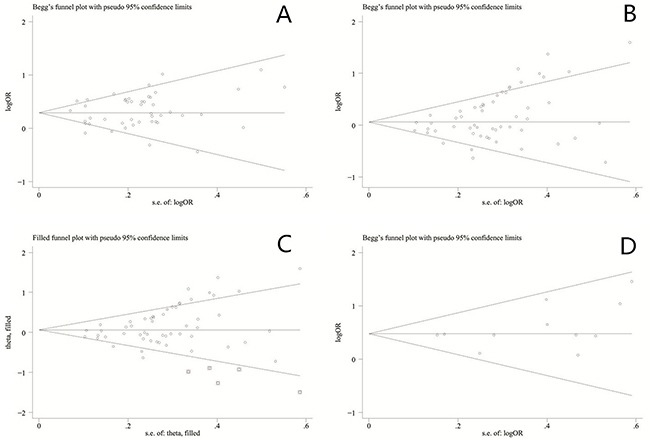
**A**. Funnel plot of GSTM1 gene polymorphism and bladder cancer risk. **B**. Funnel plot of GSTT1 gene polymorphism and bladder cancer risk. **C**. Funnel plot of GSTT1 gene polymorphism and bladder cancer risk (Trim and fill test). **D**. Funnel plot of GSTM1/GSTT1 double-null gene polymorphism and bladder cancer risk.

The funnel plot for the relationship between GSTM1 null genotype and bladder cancer susceptibility is shown in Figure 5A. The P values for Begg's and Egger's tests were 0.44 and 0.42, respectively. The results did not reveal any evidence of publication bias in this meta-analysis.

The funnel plot for the relationship between GSTT1 null genotype and bladder cancer susceptibility is shown in Figure 5B. The P values for Begg's and Egger's tests were <0.01 and 0.02, respectively. The results revealed that the publication bias was significant. Five studies were required to make the plot symmetrical (trim and fill method) (Figure 5C). Moreover, LogOR and its 95%CI altered significantly after performed trim and fill method.

The funnel plot for the relationship between GSTM1/GSTT1 double-null genotype and bladder cancer susceptibility is shown in Figure 5D. The P values for Begg's and Egger's tests were 0.35 and 0.20, respectively. The results did not reveal any evidence of publication bias in this meta-analysis.

## DISCUSSION

Both environmental and genetic factors are important in bladder carcinogenesis; however, the exact mechanisms remain unknown. Polymorphisms in GSTs may result in deficiency in GST enzyme activity and increased cancer susceptibility. Previous studies have explored the relationship between GSTM1/GSTT1 deletion polymorphisms and cancer susceptibility. However, the results were inconclusive because of different study designs, and ethnicities and lifestyles of the enrolled subjects.

To quantify the strength of the association between GSTM1/GSTT1 polymorphisms and bladder cancer risk, we performed a meta-analysis of 63 studies. GSTM1 functions in the detoxification of benzene oxide to s-phenylmercapturic acid [76], and its deletion is responsible for the deficiency in key enzyme activity. Our results show that the GSTM1 null genotype is associated with increased bladder cancer risk. GSTT1 also functions in the detoxification of various carcinogens [76]. Our study demonstrates that GSTT1 null genotype is associated with increased bladder cancer risk. The null genotype of both GSTM1 and GSTT1 is also associated with increased risk of bladder cancer.

Regarding the different ethnicities, our results suggest that the GSTM1 null genotype is associated with the elevated risk of bladder cancer in Caucasians and Asians. A similar relationship was found between the GSTT1 null genotype and bladder cancer risk in Caucasians. In addition, our results indicate that the GSTM1/GSTT1 double-null genotype is associated with elevated risk of bladder cancer in Caucasians, Asians, and Africans. This discrepancy could be explained by different effects of the GSTM1 and GSTT1 polymorphisms on bladder cancer susceptibility in different ethnic groups. Alternatively, the sample size of Asians and Africans studies might have been small, which would result in an inadequate statistical power to identify a statistically significant effect or generate a fluctuated risk estimate [77]. Stratified analyses of population-based association study showed a significant association of increased bladder cancer risk with GSTM1 null and GSTM1/GSTT1 double-null in HB studies and PB studies. However, this statistical significance was not found between GSTT1 null genotype and bladder cancer risk in HB studies and PB studies. This suggests that the role of GSTT1 deletion on bladder cancer susceptibility is mainly influenced by the different control individuals. It is well known that smoking is one of the main independent risk factors for bladder cancer [78]. After stratification by smoking status, the GSTM1 deletion was associated with an increased bladder cancer risk in both smokers and nonsmokers. However, no statistical significance was found between GSTT1 deletion and smoking status. Non-smokers with GSTM1/GSTT1 double-null genotype had an elevated bladder cancer risk. These inconsistent data may indicate the different GSTs interactions resulting in joint action. The different study weight dictated by study size may also influence the result.

Several limitations should be noted in this meta-analysis. First, several small sample size studies were included in our analysis. However, we also included some large sample size studies. Second, significant heterogeneity between trials was observed in the analysis. This heterogeneity may derive from the differences in ethnicity, population-based or individual lifestyles. Third, we detected a publication bias when we analyzed the association between GSTT1 polymorphism and bladder cancer risk. It is probably because only published and English language papers were enrolled in this study.

In summary, our meta-analysis study indicates that GSTM1 null, GSTT1 null, and GSTM1/GSTT1 double-null genotypes are associated with increased bladder cancer risk. Further high-quality large scale epidemiological studies should be performed to verify the current conclusions.

## MATERIALS AND METHODS

### Publication search

According to the PRISMA guidelines [79], we performed a systematic literature search of Pubmed, Web of Knowledge, and the Cochrane Central Search Library (on May 15, 2016). Key words used included glutathione S-transferase M1, GSTM1, glutathione S-transferase T 1, GSTT1, bladder, cancer, carcinoma, and tumor. All abstracts and review studies on this topic were reviewed. Reference lists of review studies were searched by hand.

### Inclusion and exclusion criteria

The eligible studies had to meet the following inclusion criteria: (1) The studies had to evaluate the association between GSTM1 or GSTT1 polymorphism and bladder cancer risk; (2) The report contained key information that could yield odds ratio (OR) by the data provided; (3) Studies were published in English; and (4) Conference abstracts, reviews and unpublished reports were not included. Duplicate or insufficient reports were excluded. Figure 1 shows the procedure of identifying and selecting studies.

### Data extraction

Two authors extracted the data from the eligible articles according to the inclusion criteria. Any disagreement was resolved during a discussion with a third author. The literature data were extracted individually. OR and its 95% confidence intervals (CI) were used to estimated the association between the GSTM1 or GSTT1 null polymorphism and bladder cancer susceptibility. We evaluated the quality of studies using the Newcastle–Ottawa Scale. (http://www.ohri.ca/programs/clinical_epidemiology/oxford.asp) Scores 7 to 9 were defined as a high quality study, and a score <7 as a low quality study.

### Statistical analyses

A meta-analysis was performed to reveal the association between GSTM1/GSTT1 deletion polymorphisms and bladder cancer susceptibility. Subgroup analyses were performed on different ethnicity, population-based and smoking status. Statistical heterogeneity was assessed using a formal Q-statistic as well as I^2^ [80], P<0.05 was considered statistically significant. A fixed-effect model was used when no heterogeneity was found; otherwise, the random-effect model was used to calculate pooled ORs. To validate the stability of results in this meta-analysis, a sensitivity analysis was carried out by sequential omitting each study and analyzing whether the significance of ORs was influenced excessively by omitting the study. Publication bias was evaluated by Egger's regression asymmetry test [81], and Begg's adjusted rank correlation test [82]; P<0.05 was considered statistically significant. All analyses were performed using the STATA 11.0 statistical software (Stata Corp, College Station, TX, USA).
